# Mitral valve transcatheter edge-to-edge repair as a bridge to treat aortic dissecting aneurysm in a case of Marfan syndrome: a case report

**DOI:** 10.1093/ehjcr/ytae338

**Published:** 2024-07-10

**Authors:** Takanori Kawamoto, Tomohito Kogure, Chihiro Koyanagi, Kyomi Ashihara, Junichi Yamaguchi

**Affiliations:** Department of Cardiology, Tokyo Women’s Medical University, 162-8666, 8-1 Kawada-cho, Shinjuku-ku, Tokyo, Japan; Department of Cardiology, Tokyo Women’s Medical University, 162-8666, 8-1 Kawada-cho, Shinjuku-ku, Tokyo, Japan; Department of Cardiology, Tokyo Women’s Medical University, 162-8666, 8-1 Kawada-cho, Shinjuku-ku, Tokyo, Japan; Department of Cardiology, Tokyo Women’s Medical University, 162-8666, 8-1 Kawada-cho, Shinjuku-ku, Tokyo, Japan; Department of Cardiology, Tokyo Women’s Medical University, 162-8666, 8-1 Kawada-cho, Shinjuku-ku, Tokyo, Japan

**Keywords:** Case report, Marfan syndrome, Mitral valve regurgitation, Transcatheter edge-to-edge repair for mitral valve, Dissecting aortic aneurysm, Bridging therapy

## Abstract

**Background:**

Marfan syndrome is an inherited disorder that manifests with various cardiovascular conditions. This case report discusses a patient with Marfan syndrome presenting with concurrent dissecting aortic aneurysm and acute mitral valve regurgitation (MR), exploring treatment strategies for this unique case.

**Case summary:**

A 57-year-old man diagnosed with Marfan syndrome presented with progressive dyspnoea and awareness of orthopnoea. Acute heart failure (HF) due to acute MR associated with chordae rupture was diagnosed. However, contrast-enhanced CT revealed the coexistence of a massive dissecting aortic aneurysm, indicating surgical intervention. The dissecting aortic aneurysm extended over a large area. Given the high risk of simultaneous surgery with the mitral valve, a staged approach was adopted. Mitral valve transcatheter edge-to-edge repair (MV-TEER) was performed as the initial step to reduce the perioperative HF risk, followed by a planned two-stage surgery for the dissecting aortic aneurysm. This strategy effectively facilitated surgical intervention for the dissecting aortic aneurysm in the chronic phase after MV-TEER.

**Discussion:**

Several reports showed the effectiveness of MV-TEER in cases of degenerative MR where surgical operation carries a high risk, but case report of MV-TEER in Marfan syndrome is rare. In recent years, the effectiveness of MV-TEER has also been reported as a ‘bridge therapy’ for heart transplantation. Mitral valve transcatheter edge-to-edge repair is considered a potential option to serve as a bridge to other invasive intervention.

**Learning points**

- Mitral valve insufficiency in Marfan syndrome, valve fragility is a concern at the time of surgical intervention. The outcomes of mitral valve transcatheter edge-to-edge repair in Marfan syndrome are not clear.
- It is useful to perform mitral valve transcatheter edge-to-edge repair in heart failure patients who are considered for heart transplantation, in the purpose of waiting transplantation with stable condition or deferring it.
- In cases of Marfan syndrome with coexisting conditions that pose a high risk for surgical treatment, performing mitral valve transcatheter edge-to-edge repair for mitral regurgitation might reduce the risk of heart failure, enabling sequential treatment for other coexisting conditions as well.

## Introduction

Marfan syndrome is a systemic connective tissue dysplasia of autosomal dominant inheritance associated with cardiovascular disease,^1^ including mitral regurgitation (MR), aortic root enlargement, aortic aneurysm, and aortic dissection. The aetiology of MR in patients with Marfan syndrome may be due to myxomatous degeneration of the valve leaflets; thus, valve fragility is a concern during surgical intervention. Although optimal surgical treatment of MR in Marfan syndrome (valve repair vs. replacement) has been controversial because the underlying connective tissue defect might compromise repair longevity,^2^ surgical mitral valve repair (SMVr) has been reported to have a better prognosis.^3^ However, mitral valve transcatheter edge-to-edge repair of mitral valve (MV-TEER) in patients with Marfan syndrome has been rarely reported.

## Summary figure

**Table:** 

Date	Event
Day 0: hospital admission	Hospital admission due to progressive dyspnoea and awareness of orthopnoea and diagnosed with heart failure.
Week 1	Severe mitral valve regurgitation was suspected from auscultation and confirmed by echocardiography, treated with diuretics and inotropic agents in acute phase. Then, heart failure was compensated.
Week 2	After the compensated of heart failure, transoesophageal echocardiography showed severe mitral regurgitation with ruptured chodae and it was supposed aetiology of heart failure. Contrast-enhanced computed tomography shows concomitant of large dissecting aortic aneurysm in thoracic aorta to abdominal aorta.
Week 3	Transferred to our hospital and treatment strategies were discussed in heart team. Then, we decided to treat MR first with MV-TEER, treat dissecting aortic aneurysm second with surgery.
Week 4	Performed MV-TEER and MR was reduced mild to moderate range. After that, cardiac condition was improved and stabilized.
Week 8	Performed surgery for dissecting aortic aneurysm and there was no peri-procedural complication including heart failure.

## Case presentation

**Figure 1:**
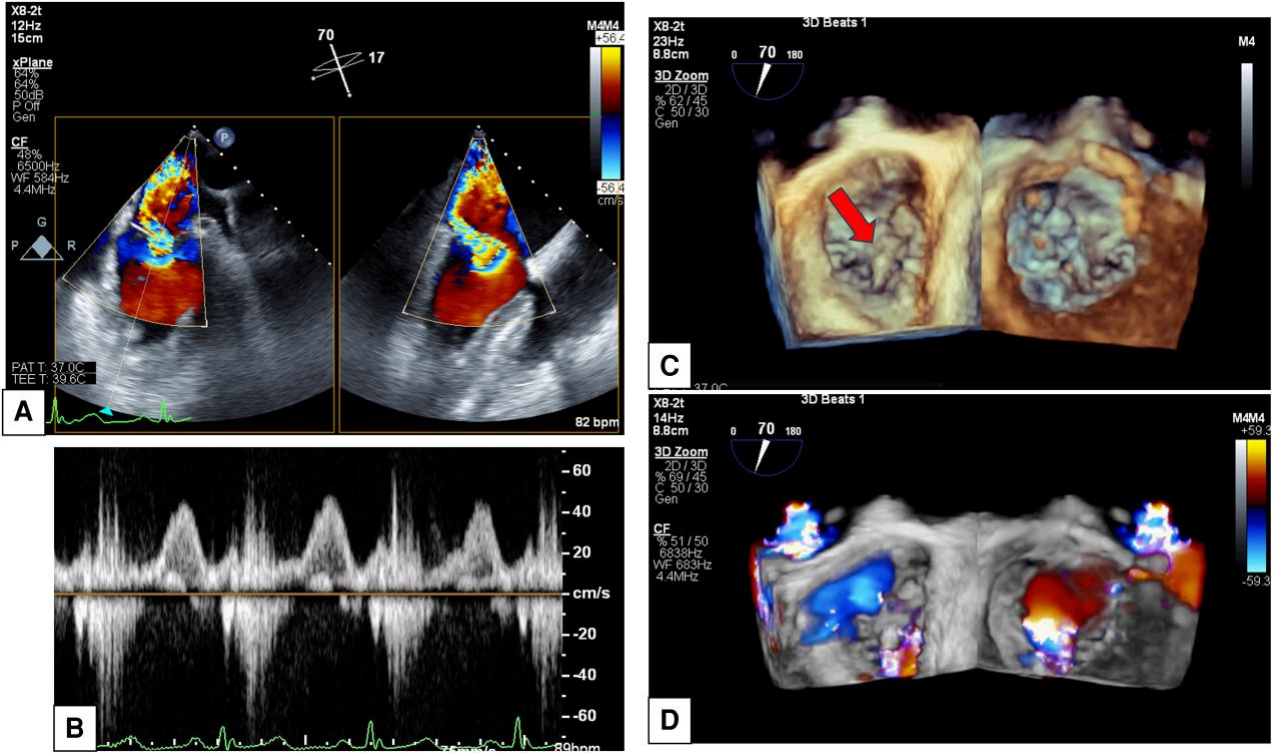
TEE findings. TEE reveals AML2 prolapse with ruptured chordae and severe regurgitation from the centre of the mitral valve. (*A*) Mitral commissural view and left ventricle and aorta long axis view. A deviated MR jet is observed originating from the prolapsed AML2. (*B*) Pulse wave Doppler findings in the left superior pulmonary vein. Pulmonary vein systolic flow reversal and finding of the S-wave being lower than the D-wave are observed. (*C*) Three-dimensional of mitral valve view from the left atrium and left ventricle. Prolapsed AML2 is observed (red arrow). (*D*) Three-dimensional of mitral valve view from the left atrium and left ventricle. Regurgitation flow from the prolapsed AML2 is confirmed, and flow convergence is observed on the left ventricular side.

**Figure 2:**
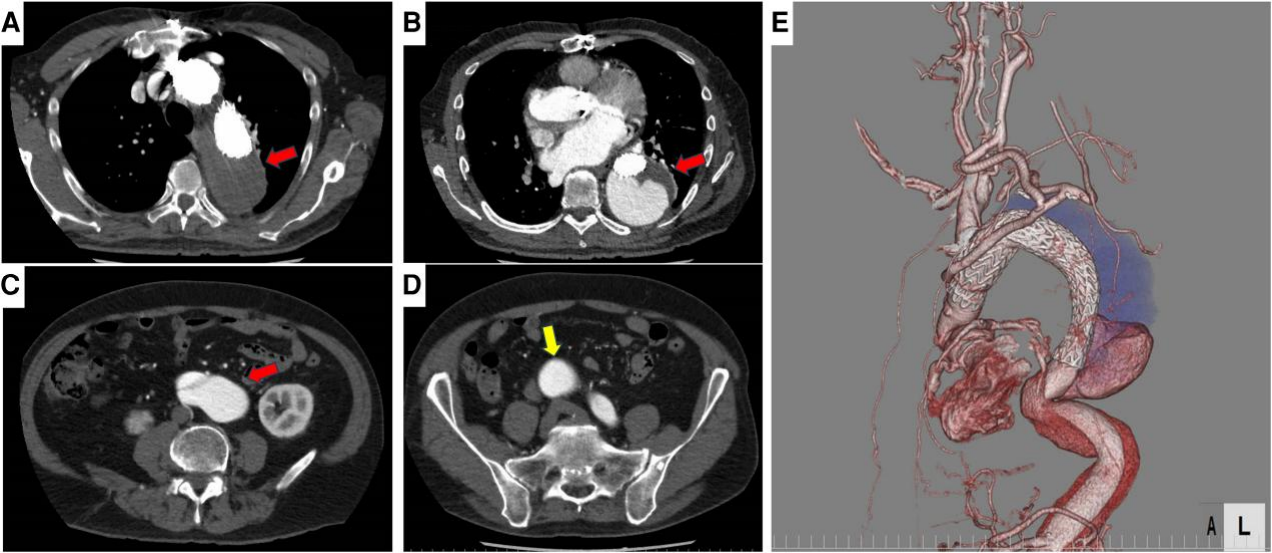
CT scan findings of dissecting aortic aneurysm. The contrast CT scan shows a thoracoabdominal dissecting aortic aneurysm with a partial false lumen opening (71 mm of maximum short diameter), and exhibiting a saccular shape (red arrow in panels *A–C*). There are residual abdominal aorta dissection and a right common iliac aneurysm (yellow arrow in panel *D*). The aortic arch is repaired with a stent graft. (*A*) Cross-section of the aortic arch, (*B*) cross-section of the thoracic descending aorta, (*C*) cross-section of the infrarenal aorta, (*D*) cross-section of the bifurcation of the common iliac artery, (*E*) 3D-CT angiography at chest level.

**Figure 3:**
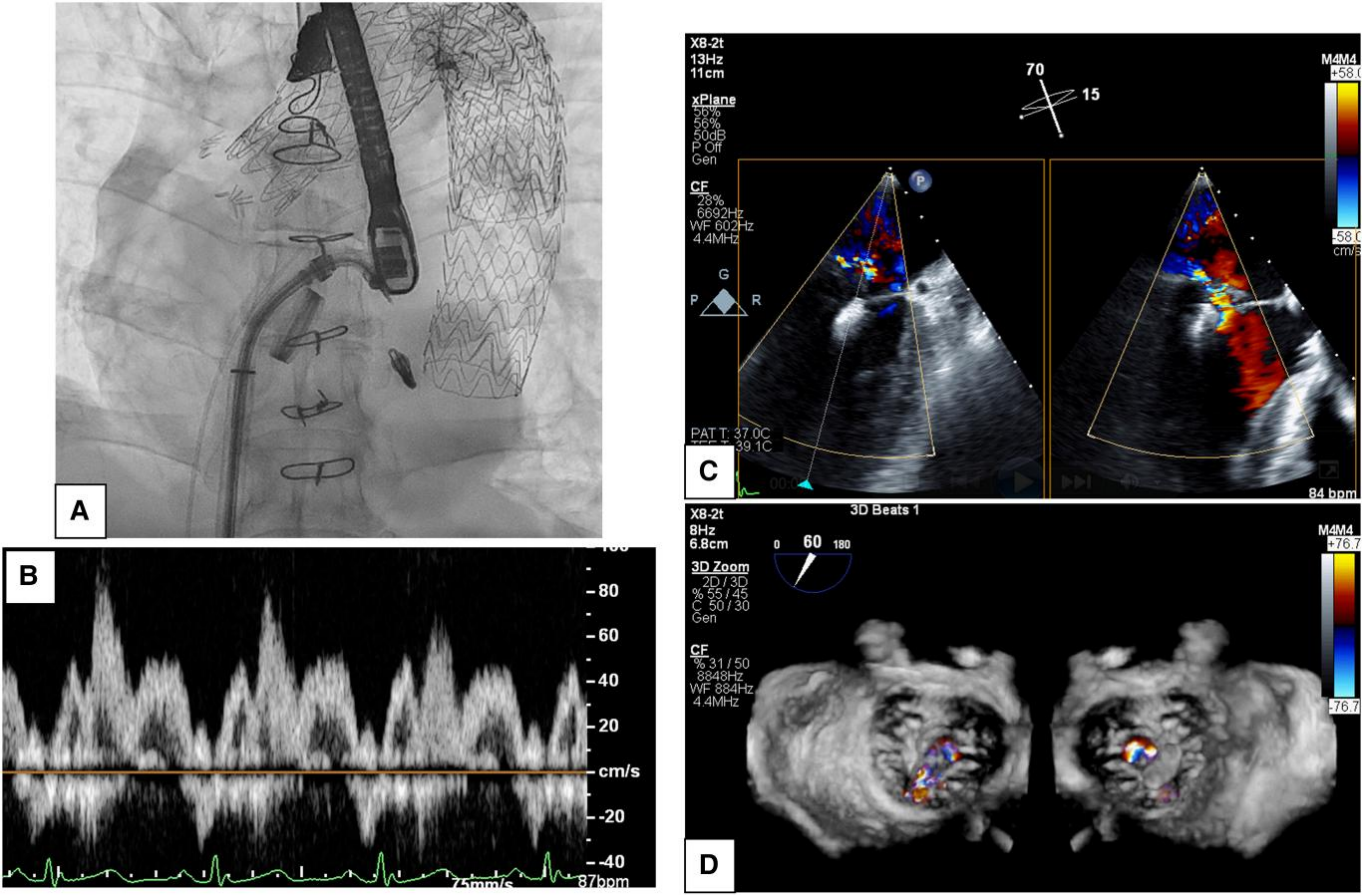
Intraprocedural findings of radiography and transoesophageal echocardiography. After the placement of the clip, mitral regurgitation is reduced but persists after single clip due to strain on the valve, resulting in a new indentation. (*A*) Fluoroscopic image after the placement of the clip. (*B*) Post-procedural pulse wave Doppler findings of the left superior pulmonary vein. Pulmonary vein systolic flow reversal is vanished and the inversion in the height of the S-wave and the D-wave is observed. (*C*) Mitral commissural view and left ventricle and aorta long axis view in TEE. Mitral regurgitation is reduced and remains mild-moderate regurgitation with placement of single clip. (*D*) Coloured three-dimensional of mitral valve view from left atrium and left ventricle on TEE. Clip is placed at the centre of AML2 and PML2 and a new indentation has occurred on the medial side of the clip, resulting in residual regurgitation from the same area.

A 57-year-old man diagnosed with known Marfan syndrome presented to a peripheral hospital with progressive dyspnoea and awareness of orthopnoea. His medical history included previous aortic dissection surgery (Bentall procedure with 25 mm CarboMedics Valsalva conduit and 28 mm LivaNova graft; total arch replacement with 26 mm four-branched J-graft, Japan Lifeline; and thoracic endovascular aortic repair with TAG 28 × 20 mm GORE) and post-operative residual thoracoabdominal dissecting aortic aneurysm (maximum diameter, 48 mm) 3 years ago. On admission, he exhibited systolic apical heart murmur and wheezing in the bilateral lung. Chest radiography showed cardiomegaly and pulmonary oedema. An electrocardiogram revealed sinus rhythm with left axis deviation, without ST changes, and a heart rate of 120 beats per minute. Laboratory test results showed: blood count, within normal range; high creatinine levels (1.3 mg/dL); mild liver dysfunction (aspartate aminotransferase, 42 U/L and alanine aminotransferase, 50 U/L); and elevated brain natriuretic peptide level (400 pg/mL). He was diagnosed with congestive heart failure (HF) and hospitalized. Diuretics and additional medications of inotropic agent compensated for HF. Post-thoracotomy yielded unclear results of transthoracic echocardiography (TTE); transoesophageal echocardiography (TEE) was performed and severe MR by anterior mitral leaflet 2 (AML2) prolapse with chordae rupture was recognized (*Figure 1* and Supplementary material online, *Video S1*). Mitral regurgitation flow into all four pulmonary veins occurred and the diastolic wave (D-wave) height was higher compared with systolic wave (S-wave) height (*Figure 1B*). The mitral valve pressure gradient was 2 mmHg and the mitral valve area was calculated (7.58 cm^2^) by three-dimensional planimetry. The lengths of AML2 and posterior mitral leaflet 2 (PML2) were 27 and 15 mm, respectively. Flail-gap between AML2 and PML2 was 9 mm. Initially, SMVr was planned. However, contrast-enhanced computed tomography (CT) showed prominent enlargement of the thoracoabdominal dissecting aortic aneurysm with a diameter of 71 mm without peripheral malperfusion and right common iliac artery aneurysm with a diameter of 41 mm (*Figure 2*). Simultaneous surgery for MR and dissection aortic aneurysm was ruled out because vascular access to the cardiopulmonary bypass (CPB) machine was not possible due to the dissecting aortic aneurysm and severe HF. The patient was transferred to our hospital for treatment, showing a state of New York Heart Association (NYHA) class Ⅲ with low blood pressure (systolic blood pressure, 90 mmHg) without inotropic agents use. Coronary CT scan and dual single photon emission CT excluded coronary artery disease. Transthoracic echocardiography confirmed severe MR due to mitral valve AML2 prolapse (volumetric measurements showed regurgitant volume (RV) of 136 mL and regurgitant fraction (RF) of 66%, effective regurgitant orifice (ERO) 87 mm^2^, proximal iso velocity hemispheric surface area radius 13 mm, RV of 114 mL, RF of 55%, ERO 73 mm^2^). The mitral valve area was calculated (8.8 cm^2^) by two-dimensional planimetry. The artificial aortic valve was functioning; left ventricular ejection fraction was 66% without severe left ventricle dilatation. There was no myxomatous degeneration of the valve leaflets. The case was discussed with our heart team. The symptoms persisted despite the maximum HF treatment possible in this case, including oral administration of furosemide (20 mg), candesartan (2.0 mg), and spironolactone (25 mg). The surgical risk was intermediate to high (Euro score Ⅱ, 3.83% and STS score, 3.73% for SMVr without considering about coexistence of aortic aneurysm). Open-heart surgery was challenging due to the inability to use CPB devices, and the mitral valve morphology in this case was considered suitable for MV-TEER implementation,^4^ focusing solely on cardiac function and the degree of mitral valve regurgitation and excluding the fact that the patient had Marfan syndrome. We decided to proceed with MV-TEER initially and perform aortic aneurysm surgery in the chronic phase after improvement in HF. We had conveyed the conference content to the patient who understood and requested the treatment plan. We performed MV-TEER under general anaesthesia with intubation. The MV-TEER procedure was performed with single clip placement (MitraClip™ G4 XTW Abbott) grasping the centre of AML2 and PML2 (see Supplementary material online, *Video S2*). Intraprocedural TEE showed that MR severity was reduced to mild to moderate (*Figure 3* and Supplementary material online, *Video S3*), and mitral valve pressure gradient changed (2–3 mmHg). Although MR remained and the mitral valve leaflets were long and wide enough to allow a second clip placement, we terminated the procedure upon confirming disappearance of regurgitation into the pulmonary vein and alterations in pulmonary venous blood flow pattern (S-wave height > D-wave height) (*Figure 3B*). The left atrial pressure decreased from 34 to 15 mmHg in the systolic period. Heart failure symptoms improved after MV-TEER. One month after the MV-TEER, replacement surgery for the thoracoabdominal aorta was performed; the patient progressed well without complications. Post-operative medication comprised only warfarin (4 mg) and azilsartan (20 mg), without HF development. At one and a half years post-MV-TEER procedure, the patient has continued to attend outpatient appointments at our hospital. Although moderate residual MR was observed on TTE, there has been no HF recurrence; he maintained NYHA class Ⅰ status. If further MR progression or HF recurrence was observed, surgical intervention for MR would be considered.

## Discussion

Both MR and aortic dissection or aneurysms are common complications in patients with Marfan syndrome. Complicated cardiovascular disease is critical in determining prognosis.^5,6^ Several reports showed the effectiveness of MV-TEER in cases of degenerative MR wherein surgical operation carries a high risk.^7^ In cases of acute MR, particularly due to chordae rupture, HF progresses immediately and requires intervention. However, such cases represent with high surgical risk, wherein surgery is considered challenging. The effectiveness and safety of MV-TEER in cases of acute MR have been reported.^8^ However, the effectiveness and safety of MV-TEER in such cases of Marfan syndrome as the underlying condition are not clear. Additionally, we were concerned about the risk of mitral valve damage due to tissue fragility. Mitral valve transcatheter edge-to-edge repair in patients with Marfan syndrome with acute MR coexistence of enlarged dissecting aortic aneurysm is not reported. In cases such as this one with life-threatening comorbidities, MV-TEER can be a treatment option. In cases of Marfan syndrome, the coexistence rate of MR and aortic aneurysm is high,^5,6^ and tissues are fragile. Therefore, we finished the procedure with a single clip placement. Although residual MR has been reported to be a harmful prognostic factor after MV-TEER,^9^ we allowed residual MR because reducing the risk of surgical intervention for aortic dissecting aneurysm was essential in this patient. If treatment of the aortic aneurysm was possible, we suggest that surgical intervention for the mitral valve would also be feasible in the event of worsening MR. Good clinical outcomes of MV-TEER as a bridge to heart transplantation have been reported.^10^ We consider that in this case, MV-TEER could also serve as a bridge if surgical intervention for the mitral valve is required in the future, after aortic surgery. Mitral valve transcatheter edge-to-edge repair may serve as a bridge to other invasive intervention.

## Conclusion

Mitral valve transcatheter edge-to-edge repair could effectively treat MR with HF in a patient with Marfan syndrome. Considering the risk of comorbidity, it could be a treatment option as a bridge therapy for treatment of further life-threatening conditions or for intervention for mitral valve disease exacerbated in the long-term period after MV-TEER.

## Supplementary Material

ytae338_Supplementary_Data

## Data Availability

The data underlying this article will be shared on reasonable request to the corresponding author.

## Lead author biography

Title/position: medical assistant (physician). Affiliation: Tokyo Women’s Medical University. Responsibilities: Overseeing patient care, performing coronary intervention, peripheral artery intervention, transcatheter structural heart disease intervention, echocardiography, conducting research on cardiovascular diseases.

## Supplementary material

**Consent:** Written informed consent for the publication of clinical details and clinical images was obtained from the patient, compliant with COPE guidelines. Permission to reproduce material from other sources.

**Funding:** No funding was provided for this paper.
